# How challenge and hindrance research stressors affect university teachers’ research performance: the mediating role of researcher identity and the moderating role of research environment

**DOI:** 10.3389/fpsyg.2026.1786936

**Published:** 2026-05-11

**Authors:** Zhe Zhang, Yuming Wang, Minxiao Zheng, Xinbo Wan, Yaqin Bian

**Affiliations:** 1 School of Education, Huazhong University of Science and Technology, Wuhan, China; 2 School of Electronic Information and Communications, Huazhong University of Science and Technology, Wuhan, China; 3 School of Education, Jianghan University, Wuhan, China; 4 School of Educational Science, Hubei Normal University, Huangshi, China; 5 School of Education, Central China Normal University, Wuhan, China

**Keywords:** challenge-related stressors, hindrance-related stressors, research environment, research performance, researcher identity

## Abstract

**Objective:**

Under the national strategies of “Double First-Class” construction and innovation-driven development, research performance has become a critical metric for evaluating universities’ core competitiveness and faculty members’ academic contributions. However, university teachers generally face increasing research pressure in their pursuit of high performance, and traditional views have often simplistically regarded such pressure as a purely negative factor, thereby overlooking its potential positive motivational effects. To address this research gap, the present study investigated how challenge and hindrance stressors affect university teachers’ research performance, with a focus on the mediating role of researcher identity and the moderating role of the research environment.

**Methods:**

A cross-sectional survey was conducted among 560 university teachers in China. Descriptive statistics as well as reliability and validity tests were performed using SPSS 25.0. A structural equation model was constructed using AMOS 26.0 to test the mediating effect of research role identity, and hierarchical regression analysis was employed to examine the moderating effect of the research environment.

**Results:**

The results showed that both types of stressors influenced research performance, with challenge stressors and hindrance stressors exerting opposite effects. Research role identity partially mediated the relationships between both types of stressors and research performance. Moreover, the quality of the research environment not only directly promoted research performance but also significantly moderated the relationship between research role identity and research performance; specifically, a high-quality research environment strengthened the facilitative effect of role identity on performance.

**Conclusion:**

Theoretically, this study reveals the dynamic adaptive mechanism between research pressure and research performance, extending the challenge–hindrance stressor framework to the higher education context. Practically, it recommends that universities differentiate the nature of stressors, strengthen the cultivation of teachers’ research role identity, and improve research environment support systems to facilitate the positive transformation of research pressure into performance improvement.

## 1 Introduction

In the 21st century, the global economy is driven by knowledge and innovation. Countries are enhancing their core competitiveness by improving their ability of scientific and technological innovation. All major powers in the world have placed scientific and technological innovation at the top of their national development strategies. China is at a critical stage of economic transformation and upgrading. It needs to build a new development pattern and promote high-quality development by achieving high-level scientific and technological self-reliance and breaking the “bottleneck” of technology. As an important part of the national innovation system and the main force of basic research, colleges and universities are responsible for knowledge production, scientific and technological innovation and high-end personnel training, and always stand at the forefront of promoting scientific and technological progress. The level of their scientific research performance directly determines the growth rate of national knowledge stock, the possibility of technological breakthrough and the vitality of innovative culture. Scientific research performance has become a key indicator to measure the comprehensive strength of universities. Therefore, the status and performance of scientific research work of university teachers have received unprecedented attention. In order to better stimulate the innovation potential and work enthusiasm of scientific research personnel, major universities across the country have carried out a series of personnel and scientific research reform. Some universities have implemented the faculty appointment system on a large scale and adopted employment systems as well as salary and professional title evaluation standards that take research performance as the primary criterion (Wang and Zhang, 2026; Zhang et al., 2025). Under such systems, faculty members are typically hired on fixed-term contracts (e.g., 3–6 years) and must meet clearly defined research output targets—such as publishing a specified number of high-impact journal articles (e.g., SCI/SSCI papers) or securing national-level research grants (e.g., NSFC, NSSF)—to be eligible for contract renewal or promotion. Some schools even adopt “up or go” and other types of assessment methods, whereby faculty who fail to meet performance benchmarks within the probationary period are dismissed rather than granted tenure or continued employment (Gu et al., 2010; Feng, 2012). These practices are often accompanied by rigorous annual performance reviews, performance-based salary adjustments, and the linking of research outcomes to promotion timelines. Consequently, such a high-stakes, performance-oriented competitive environment has profoundly transformed the academic career ecology of university teachers, where in research pressure has become a universal and normalized experience.

However, how does the pervasive research pressure actually affect teachers’ research performance? Traditional perspectives and management practices tend to view pressure as a homogeneous negative psychological variable, simply linking increased pressure to adverse outcomes such as performance decline, burnout, and academic misconduct (Zhang and Liao, 2012). Although this linear, one-dimensional cognitive framework is intuitively appealing, it may obscure the complexity and inherent contradictions in the process through which pressure influences research performance. Some studies have found that not all pressure necessarily leads to negative consequences. For instance, Chinese scholars Ji Xiaoli and colleagues pointed out that external work pressure has a negative effect on university teachers’ job performance, whereas internal pressure has a facilitating effect (Ji and Chen, 2009). This complexity suggests that treating research pressure as a “black box” can no longer satisfy the demands of theoretical development or management practice. Researchers must move beyond a generic understanding of pressure and adopt a more nuanced theoretical perspective to distinguish how different types of stressors influence research performance.

Whether research pressure promotes or hinders research performance remains an open question. The challenge–hindrance stressor framework distinguishes two qualitatively different types of work-related demands: challenge stressors, which are perceived as opportunities for growth and achievement, and hindrance stressors, which are seen as obstacles that impede personal development (Cavanaugh et al., 2000). This framework posits that the former tend to yield positive outcomes, whereas the latter typically result in negative consequences (Podsakoff et al., 2023). Such a distinction has advanced stress research by reconciling previously inconsistent findings on the stress–performance relationship and by enabling more targeted and effective stress management strategies (LePine, 2022). Existing literature suggests that the relationship between challenge-related and hindrance-related stressors and work performance is not simply linear but there are complex mediating and moderating mechanisms (Lepine et al., 2005; Abbas and Raja, 2019; Sawhney and Michel, 2022). The two-dimensional stressor model offers a valuable analytical framework for studying the impact of research pressure on research performance among university teachers. In this framework, teachers’ research pressure is classified into two types: challenge research stressors and hindrance research stressors. Specifically, challenge stressors are hypothesized to positively predict research performance, whereas hindrance stressors are hypothesized to negatively predict it.

Research performance refers to the observable and evaluable outcomes and behavioral anifestations resulting from faculty members’ research activities over a specified period (Zhang et al., 2025). From a measurement perspective, it generally encompasses two orientations. The outcome-oriented approach evaluates performance based primarily on quantifiable research outputs, such as the number and tier of published academic papers (journal index category, impact factor), the level and funding amount of research projects, the grade of published monographs, and the level of research awards, emphasizing final products and offering advantages in comparability and operability (Nie et al., 2023; Wu et al., 2023). In contrast, the behavior-oriented approach incorporates indicators of effort, collaboration, knowledge dissemination, and academic service contributions during the research process, aiming to address the limitations of outcome-oriented evaluation—which tends to overlook research effort and process—thereby providing a more comprehensive assessment of faculty members’ research contributions (Iyengar et al., 2009; Joiner, 2009; Zhu and Wu, 2023). The outcome-oriented approach is objective and comparable but encourages short-term gains and overlooks research effort. One study argued that overly frequent and intensive quantitative assessments may easily induce short-sighted, utilitarian behaviors, leading faculty to prioritize projects that yield quick results while avoiding research that is more difficult or lengthy (Li, 2011). Another study further criticized the equation “journal tier = paper quality = academic competence = performance” as highly unreasonable, because journal evaluation indicators have inherent limitations—citation counts and impact factors often reflect hot topics in a specific period rather than the true academic value of a paper (Yu, 2012). The behavior-oriented approach focuses on effort, collaboration, and academic service, addressing many limitations of outcome-oriented evaluation. Therefore, many studies have adopted a behavior-oriented evaluation approach to address these limitations (Wang et al., 2013; Zheng et al., 2022), and the present study does so as well.

Research role identity refers to the degree to which a teacher internally accepts and values their identity as a researcher, encompassing the centrality and importance of that role in the individual’s self-concept as well as the emotional investment attached to it. Role identity theory posits that individuals define themselves partly by the social roles they occupy, and that the salience or centrality of a particular role in one’s self-concept influences their behavior, effort, and persistence in role-related activities. When a teacher strongly identifies as a researcher, they are more likely to engage in problem-solving coping strategies and seek resources to meet role demands (Callero, 1985; Summers et al., 1995; Anglin et al., 2022). Therefore, in the context of our study, role identity theory suggests that research stressors—both challenge and hindrance types—influence research performance through the psychological pathway of research role identity. Specifically, challenge stressors may strengthen research role identity by providing opportunities for achievement and recognition, while hindrance stressors may weaken it by frustrating goal progress. Transactional stress theory indicates that the transition from external stressors to psychological, physiological, and behavioral responses is influenced by environmental factors (Kraimer et al., 2022). As a critical situational variable, the research environment can influence research performance by enhancing the positive effects of challenge-related stressors and mitigating the negative effects of hindrance-related stressors. However, the moderating function of the research environment between stressors and research performance is complex, and the academic community has not reached a consensus on the relationship between the two (Mazzola and Disselhorst, 2019). Some studies have found that external factors, such as supervisor style, can moderate the relationships among stressors, role identity, and academic passion. For instance, inclusive supervisor mentoring, as a supportive organizational context, can suppress the negative effect of hindrance research stressors on doctoral students’ research role identity, thereby weakening the indirect negative influence of hindrance research stressors on academic passion through research role identity (Liu and Gao, 2024). In contrast, abusive supervisor mentoring style has been shown to have a significant positive moderating effect on the relationship between challenge stressors and research performance, while having no significant effect on the relationship between hindrance stressors and research performance (Chang, 2021). These findings suggest that the research environment may affect faculty research performance either by amplifying the benefits of challenge stressors or by mitigating the negative effects of hindrance stressors. However, the specific pathways through which the research environment moderates the relationship between research stressors and research performance among university faculty remain largely under-investigated and require further empirical testing.

All in all, though previous studies have provided valuable insights, several important gaps remain. First, while the challenge–hindrance stressor framework has been extensively tested in corporate and general employee samples, its applicability to university teachers in the Chinese higher education context—particularly under the pressures of the “Double First-Class” initiative and “publish or perish”—remains limited. Most existing studies have treated research pressure as a homogeneous construct, overlooking the differential effects of challenge and hindrance stressors on research performance. Second, the psychological mechanisms linking research stressors to performance, especially the mediating role of research role identity, have received little empirical attention. Although role identity theory suggests that individuals with a strong researcher identity are more likely to adopt proactive coping strategies, few studies have directly tested whether research role identity transmits the effects of challenge and hindrance stressors to performance outcomes. Third, the potential moderating role of the research environment in the stressor–identity–performance chain remains largely unexplored. While transactional stress theory posits that contextual factors shape stressor–response relationships, empirical evidence on how the research environment interacts with role identity to influence research performance is scarce, particularly in the Chinese academic context. To address these gaps, drawing upon the two-dimensional framework of challenge–hindrance stressors and taking Chinese university teachers as the research subjects, this study addresses the following research questions: (1) to verify the differentiated effects of challenge and hindrance research stressors on teachers’ research performance, thereby identifying the direction and magnitude of the influence exerted by each type of stressor, providing a more nuanced understanding than prior studies that treated pressure as a unidimensional variable; (2) to examine the mediating role of research role identity in the relationship between the two types of stressors and research performance, thereby uncovering the psychological pathway through which research stressors influence performance; (3) to assess the moderating role of the research environment in the stressor–performance pathway, extending the challenge–hindrance framework by demonstrating how contextual factors can amplify or buffer the effects of stressors. By focusing on Chinese university teachers—a population under intense research pressure from national policies and institutional rankings—this study enriches the theoretical application of the challenge–hindrance model in the higher education sector and provides actionable empirical evidence for research management and stress intervention.

## 2 Theoretical basis and research hypotheses

### 2.1 The relationship between challenge– and hindrance-related stressors and research performance

Research stress, as an important contextual cue, not only highlights the challenges faced in research activities but also influences teachers’ assessments of the cost required to overcome these challenges and expected benefits, affecting their construction of researcher identity (Li et al., 2018). The Stress Interaction Theory posits that individuals perceive and evaluate stressors based on specific environment and their own coping resources (Kraimer et al., 2022). Building on this, Cavanaugh et al. proposed a two-dimensional framework model of challenge–hindrance stressors to examine the different impacts of various environments on individual cognitive assessments. Challenge-related stressors refer to stressors that have potential rewards for individual growth and development, such as time pressure, workload, work responsibilities, and complexity. These are task-deriving stressors that typically have a positive impact on individuals’ psychological cognition and behaviors. In contrast, hindrance-related stressors are those that individuals perceive as difficult to overcome and that impede goal achievement and personal development, such as role conflict, role ambiguity, organization politics and job insecurity. These are relationship-deriving stressors that often bring negative effects to individuals’ emotions, cognition and behaviors (Cavanaugh et al., 2000). Boswell et al. (2009) pointed out that coping with challenge-related stressors is considered motivational, providing individuals with learning opportunities, potential benefits as well as positive emotional experiences. Such stressors incentivize teachers and light their passion, leading them to invest more in research work and achieve research progress by continuously overcoming challenges. Hindrance-related stressors is positively associated with anxiety, causing the depletion of cognitive resources and emotional exhaustion, undermining teachers’ self-efficacy and motivation (Yao and Ma, 2021) and detrimental to growth. As such, the hypotheses are proposed:

*H1a*: Challenge-related stressors have significant positive correlation with teachers’ research performance.

*H1b*: Hindrance-related stressors have significant negative correlation with teachers’ research performance.

### 2.2 The mediating role of researcher identity

According to role identity theory, role identity is an individual’s self-perception and categorization within a specific role, influenced by environment factors (Callero, 1985; Summers et al., 1995; Anglin et al., 2022). In teachers’ research work, stressors, as a prevalent external environment factor, affect teachers’ perceptions of their own research capabilities, thereby influencing their construction of researcher identity. Challenge-related stressors, such as high workloads, tight deadlines, and demanding projects, are generally viewed as opportunities for growth and achievement. These stressors can enhance researcher identity by fostering a sense of competence, achievement, and professional pride. When researchers successfully cope with these challenges, they may increase their self-efficacy and develop a stronger identification with their research roles (Lepine et al., 2005). Similarly, Bakker and Demerouti reported that challenging tasks can enhance employees’ engagement and their identification with their work roles (Bakker and Demerouti, 2007). On the other hand, hindrance-related stressors, such as organization constraints, role ambiguity, and interpersonal conflicts are perceived as obstacles to achieving goals and can undermine researcher identity. These stressors often lead to frustration, anxiety, and burnout, negatively impacting researchers’ commitment and role identity. Spector and Jex (1998) found that role ambiguity and conflicts are associated with lower job satisfaction and organizational commitment, which are crucial for strong role identity. Likewise, Podsakoff et al. (2007) demonstrated that hindrance-related stressors negatively affect job performance and citizenship behavior, further undermining role identity. Based on this, the hypotheses are proposed:

*H2a*: Challenge-related stressors have significant positive correlation with teachers’ researcher identity.

*H2b*: Hindrance-related stressors have significant negative correlation with teachers’ researcher identity.

Individuals assume multiple roles within society, and thus role identity has a certain contextuality. University teachers’ researcher identity refers to the extent to which this group integrates research into their self-concept (Yin et al., 2016). The stronger an individual’s sense of role identity, the more their behavior is influenced by that identity (Albert et al., 2000). Why is this case? The internal mechanisms that affect research identity have been explored. Researcher identity can enhance researchers’ perception of the significance of their work, and work significance can promote creativity (Cohen-Meitar et al., 2009). Other studies suggest that researcher identity positively influences resilience (Perez et al., 2014), which is important in research innovation. Therefore, researcher identity has a positive effect on promoting research performance. Given this, the hypothesis is proposed:

*H3*: Researcher identity has significant positive correlation with research performance.

Role identity theory posits that role identity primarily arises from the interaction between individuals and environment, as well as from individuals’ role cognition (Ng and Feldman, 2007). The core value of role identity lies in its ability to transform external role expectations into a part of self-awareness, maintaining consistency between individuals’ behavioral attitudes and the social roles they undertake (Wang and Cheng, 2010). This implies that once teachers identify themselves as researchers, they will gain varying levels of support from research environment and achieve different degrees of research performance. Challenge-related stressors help improve research performance by enhancing researcher identity and self-efficacy. In the process of overcoming research challenges, teachers accumulate successful experiences, strengthen their confidence in their research abilities, thereby increasing their identification with their research roles and actively engaging in research. When faced with extensive hindrance-related stressors, teachers are prone to falling into self-denial and self-doubt. They may exhibit lower research self-efficacy and confidence and even question the meaning and value of research activities, appearing exhausted mentally and physically, which negatively affects their construction of researcher identity and inhibit academic passion (Pearsall et al., 2009). Therefore, researcher identity can be considered as a mediator between the research environment and research performance. According to this, the hypotheses are proposed:

*H4a*: Researcher identity mediates between challenge-related stressors and research performance.

*H4b:* Researcher identity mediates between hindrance-related stressors and research performance.

### 2.3 The moderating role of research environment

The research environment is a crucial factor influencing research performance. It encompasses multiple aspects, including material resources such as research funding and equipment, organization support such as management systems and administrative support, and psychological support such as team atmosphere and psychological counseling (Fink, 2013). The research environment is a multifaceted concept, with some researchers dividing it into soft and hard environments. The hard environment includes the working conditions provided by universities for faculty to conduct research activities, such as research facilities, research materials, and auxiliary support. The soft environment primarily refers to various policies implemented by the university to foster a strong research atmosphere, including research funding, research time, research workload, collective research consciousness, and research rewards and penalties. Overall, academic environment, development environment, and institutional environment are important components of the research environment.

Researchers have indicated that there is a complex interaction between the research environment and research stress. For example, sufficient resource support may enhance the positive effects of challenge-related stressors, but the same resources may not completely offset the negative impacts of hindrance-related stressors and may even exacerbate the sense of pressure in certain cases. Amabile (2017) emphasize that environments which foster progress and provide positive feedback can significantly enhance motivation and performance. Specifically, adequate research resources and supportive management systems create the necessary conditions and safeguards for teachers, enabling them to better tap into their potential when facing challenging pressures, thereby improving their research performance. However, the relationship between environment and stress varies in different research environments. Highly supportive environments can reduce the perceived impact of challenge-related stressors because necessary resources are readily available, making challenges easier to manage. In contrast, in less supportive environments, researchers may rely more on their ability to overcome challenge-related stress, which can either enhance their resilience or lead to greater stress.

Studies have shown that a supportive research environment can mitigate the negative effects of hindrance-related stressors. Rhoades and Eisenberger (2002) found that perceived organization support enhances employees’ emotional attachment to their roles and organizations, alleviating the adverse effects of hindrance-related stress. Podsakoff et al. (2007) discovered that a supportive environment reduces the detrimental impact of hindrance-related stressors on job performance, although this effect is not exterminate. According to Conservation of Resources Theory, even in resource-rich environments, continuous exposure to hindrance-related stressors can lead to exhaustion (Hobfoll, 1989). This is because high demands and expectations can exacerbate the stress brought by hindrance-related stressors, resulting in greater emotional and cognitive strain. In resource-rich environments, the positive effects of challenge-related stressors are more pronounced. These environments provide researchers with the necessary tools and support, improving their creativity and productivity. However, when faced with persistent hindrance-related stressors, teachers may find resources exhausted more quickly, surge in stress and decline in performance. Overall, the research environment influences individuals’ perception of research stress, affecting their research performance. Therefore, the hypotheses are proposed:

*H5a:* The research environment enhances the positive impact of challenge-related stressors on research performance.

*H5b:* The research environment intensifies the negative impact of hindrance-related stressors on research performance.

**Figure 1:**
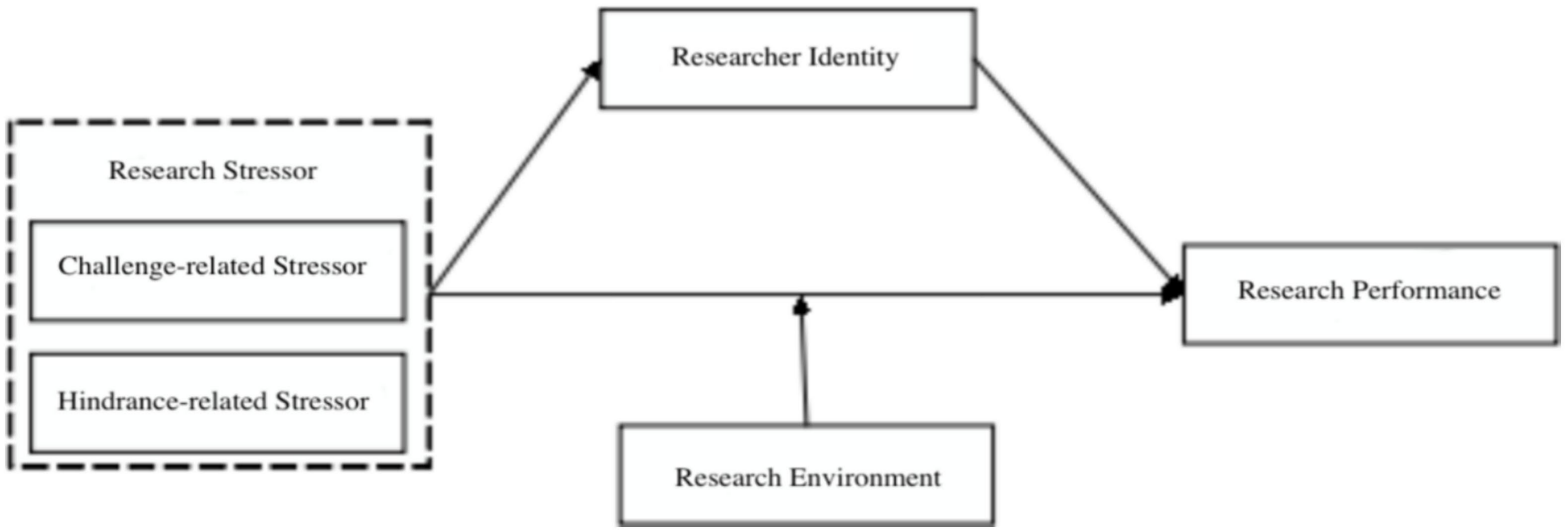
Theoretical model.

In summary, the challenge–hindrance framework distinguishes challenge stressors (perceived as growth opportunities) from hindrance stressors (perceived as obstacles). Role identity theory suggests that research role identity mediates the stressor–performance link: challenge stressors strengthen identity, while hindrance stressors weaken it. Transactional stress theory posits that the research environment moderates this process by buffering hindrance effects and amplifying challenge benefits. Integrating these theories, we propose a moderated mediation model (Figure 1): challenge stressors positively predict research performance, hindrance stressors negatively predict it, research role identity partially mediates these effects, and the research environment moderates the identity–performance relationship. The aim is to explore the intrinsic mechanisms and conditions through which research stressors affect teachers’ research performance.

## 3 Research methods

### 3.1 Research subjects

Given the specific characteristics of the target participants, a mixed sampling strategy combining convenience sampling and purposive sampling was adopted. The required sample size was determined *a priori* using G*Power 3.1.9.7. For a correlation analysis with a medium effect size (*r* = 0.30), *α* = 0.05, and power = 0.80, the minimum sample size was 84. For a multiple regression, the required sample was 92. For moderation analysis, the required sample was 77. Data were obtained from five universities located in Beijing, Wuhan, and other cities. University faculty members engaged in research activities were selected as the study participants. To ensure sample representativeness and distributional adequacy, faculty from three categories of higher education institutions—namely, “Double First-Class” comprehensive universities, “First-Class Discipline” universities, and regular undergraduate universities—were included in the survey. To mitigate the potential for self-presentation bias inherent in self-report surveys, it was explicitly stated in the questionnaire preamble that the investigation was conducted solely for academic research purposes. These responses would not be linked to any performance evaluation or reward/sanction system, and that individual information would be kept entirely confidential. Three modes of survey administration were employed: on-site distribution (faculty were invited to complete the questionnaire face-to-face), entrusted distribution via intermediaries, and online distribution (electronic questionnaires were sent to faculty email addresses). A total of 569 questionnaires were distributed, all of which were returned, yielding 560 valid responses and a valid response rate of 98.4%. The final sample of 560 valid responses exceeded all these thresholds. A *post-hoc* power analysis indicated that with *N* = 560, the achieved power was >0.99 for detecting medium-sized effects, confirming that the study is adequately powered. Detailed demographic information of the participants is presented in Table 1.

**Table 1:** Distribution of participants (*N* = 560).

Variable	Category	Number	Percentage (%)
Gender	Male	260	46.4%
	Female	300	53.6%
Age	<30	58	10.4%
	31–40	244	43.6%
	41–50	198	35.4%
	>50	60	10.7%
Academic rank	Teaching assistant	104	18.6%
	Lecturer	196	35%
	Associate professor	166	28.4%
	Professor	94	14.2%
University type	Double first-class university	354	63.2%
	First-class discipline university	96	17.1%
	Regular undergraduate university	110	19.6%
Discipline type	Science and engineering	296	52.9%
	Humanities and social sciences	264	47.1%

### 3.2 Research instruments

The assessment of research performance is based on the scale developed by Wang et al. (2014). There are totally six items in the scale, such as “I frequently participate in academic conferences.” In this study, the Cronbach’s alpha coefficient for research performance is 0.891, indicating good reliability. The Kaiser–Meyer–Olkin (KMO) measure is 0.825, and *p* < 0.001, suggesting suitability for factor analysis. Confirmatory factor analysis for structural validity yielded the following results: *χ*^2^/df = 2.23, RMR = 0.03, CFI = 0.99, TLI = 0.98, RMSEA = 0.07, indicating that the scale has good structural validity. Table 2 shows the specific contents of the questionnaire.

**Table 2:** Measurement items.

Dimension	Item no.	Item statement
Research stressors	RS1	The research assessment goals set by my college are very high.
	RS2	The amount of research tasks I need to complete is very large.
	RS3	I often feel time pressure in my research work.
	RS4	Conducting research requires me to master many research methods.
	RS5	It is difficult to explore interesting and novel research topics.
	RS6	It is difficult to achieve innovation in research results.
	RS7	High-level academic papers are very difficult to publish.
	RS8	I do not know how to become an outstanding scholar.
	RS9	It is difficult for me to find research partners with similar interests.
	RS10	My academic ability seems to have stagnated.
	RS11	Evaluations for projects, promotions, etc., are unfair, emphasizing seniority over ability.
	RS12	I have to deal with many bureaucratic procedures in my research work.
	RS13	I lack resources for conducting academic research.
	RS14	Journal review cycles are too long.
Research performance	RP1	I often attend academic conferences.
	RP2	I always actively apply for and participate in research projects.
	RP3	I strive to write high-level academic papers.
	RP4	I invest a great deal of effort in research.
	RP5	I have a certain number of high-level academic papers.
	RP6	The quantity and level of research projects I have been awarded exceed the average requirement for my position.
Research role identity	RRI1	I have a strong sense of belonging to research work.
	RRI2	Being a researcher is an important reflection of my self-identity.
	RRI3	I am a researcher.
	RRI4	Being a researcher is an important part of my self-image.
	RRI5	I feel I belong to the research field/community.
Research environment	RE1	My college offers rich academic exchange activities.
	RE2	My college has sufficient academic resources.
	RE3	My college provides smooth academic communication channels.
	RE4	My college has a sense of cooperation and team spirit.
	RE5	My college provides good guidance for academic innovation.
	RE6	My college provides opportunities for further study and training.
	RE7	My college provides a platform for peer seminars/discussions.
	RE8	My college provides financial support for further study and training.
	RE9	My college offers good career development prospects.
	RE10	My university pays attention to the growth of young faculty in all disciplines.
	RE11	My university has reward and punishment measures for faculty’s research task completion.
	RE12	I think the university’s reward/punishment measures are reasonably designed for my discipline.
	RE13	My university has clear regulations on faculty promotion.
	RE14	I think the university’s promotion system is reasonably designed for my discipline.
	RE15	My university has clear regulations on faculty salary.
	RE16	I think the university’s salary system is reasonably designed for my discipline.

The assessment of research environment is based on the scale developed by Yang Xiuxiu (Yang and Hu, 2021). It includes three dimensions: academic environment, development environment, and institutional environment, comprising a total of 16 items, such as “The university has abundant academic exchanges” (academic environment), “The university provides good career development opportunities” (development environment), and “The university has reward and punishment measures for teachers completing research tasks” (institutional environment). The Cronbach’s alpha coefficient for research environment is 0.967, indicating good reliability; KMO is 0.954, and *p* < 0.001, indicating suitability for factor analysis. Confirmatory factor analysis for structural validity showed: *χ*^2^/df = 3.09, RMR = 0.03, CFI = 0.99, TLI = 0.99, RMSEA = 0.04, demonstrating good structural validity of the scale. Table 2 shows the specific contents of the questionnaire.

The assessment of researcher identity is based on the scale developed by Robnett et al. (2015), including five items, such as “I feel a sense of belonging to my research field.” The Cronbach’s alpha coefficient for researcher identity is 0.925, indicating good reliability; KMO is 0.863, and *p* < 0.001, indicating suitability for factor analysis. Confirmatory factor analysis for structural validity resulted in: *χ*^2^/df = 2.64, RMR = 0.01, CFI = 0.99, TLI = 0.99, RMSEA = 0.07, indicating good structural validity of the scale. Table 2 shows the specific contents of the questionnaire.

The assessment of research stressors is based on the scale developed by Wang et al. (2013). All stressors fall into two categories: challenge-related stressors and hindrance-related stressors, comprising a total of 14 items, such as “I often feel time-constrained during research” (challenge-related stressors), “my academic ability perhaps comes into plateau” (hindrance-related stressors). The Cronbach’s alpha coefficients for challenge-related stressors, and hindrance-related stressors are 0.804 and 0.831, indicating good reliability; KMO measures are 0.753 and 0.829, and *p* < 0.001, indicating suitability for factor analysis. Confirmatory factor analysis for structural validity showed: *χ*^2^/df = 1.67, RMR = 0.05, CFI = 0.98, TLI = 0.96, RMSEA = 0.05, indicating good structural validity of the scale. Table 2 shows the specific contents of the questionnaire.

Additionally, existing researches have indicated that personal characteristics of teachers, such as gender, age, institutional level, academic discipline, academic title, and education background may influence research performance. Therefore, this study also takes these factors into account (Wu et al., 2023).

### 3.3 Data analysis methods

Firstly, this study utilizes SPSS 26.0 for reliability and validity testing, descriptive statistics, correlation analysis and common method bias testing. Then, to test the mediation hypotheses, AMOS 26.0 is employed for structural validity analysis of the measurement model and path analysis of the structural equation model for mediating effects, and the significance of mediating effects is tested using a estimation method of bias-corrected non-parametric percentile Bootstrap. This approach was chosen because it provides overall model fit indices and accounts for measurement error, making it appropriate for theory testing (Kelloway, 1995). Finally, to test the moderation hypothesis, hierarchical regression analysis was conducted using SPSS 25.0, as this method is straightforward for interpreting interaction effects and simple slopes (Toothaker, 1994; Hayes et al., 2012). While moderated mediation could be estimated in a single SEM model, the two-step approach is methodologically transparent and appropriate given our distinct hypotheses and sample size.

## 4 Empirical analysis

### 4.1 Common method bias test

Given that all variable data in this study are derived from respondents’ self-reports, there is a potential risk of common method bias. To mitigate it, the following approaches were employed: First, procedural controls were implemented by emphasizing anonymity, confidentiality, and the use of data solely for academic research during the data collection process. Some items were reverse-coded. Second, Harman’s single-factor test was conducted via exploratory factor analysis on all scale items. The results indicated that seven factors had eigenvalues greater than 1 before rotation, with the first common factor explaining 27.3% of the total variance, which is below the critical threshold of 40%. Furthermore, confirmatory factor analysis of the single-factor model showed poor fit indices (*χ*^2^/df = 7.276, RMR = 0.175, RMSEA = 0.150, IFI = 0.479, CFI = 0.477, GFI = 0.348). These results suggest that there is no severe common method bias in the data of this study.

### 4.2 Descriptive statistics and correlation analysis

Table 3 presents the means, standard deviations, and correlation coefficients of the variables. The data indicate that the research environment, research performance, and researcher identity are relatively high, with teachers primarily facing challenge-related stressors, which far exceed hindrance-related stressors. Additionally, the research environment is significantly positively correlated with research performance, researcher identity, and challenge-related stressors. Similarly, researcher identity and challenge-related stressors are significantly positively correlated with research environments and research performance. In contract, hindrance-related stressors are significantly negatively correlated with research environment, research performance, and researcher identity.

**Table 3:** Means, standard deviations, and correlation coefficients of variables (*N* = 560).

Variable	Mean	Standard deviation	1	2	3	4	5
Research environment	4.02	0.83	1				
Research performance	3.99	0.76	0.367**	1			
Researcher identity	3.91	0.90	0.357**	0.715**	1		
Challenge-related stressors	4.16	0.58	0.114**	0.324**	0.195**	1	
Hindrance-related stressors	3.40	0.79	−0.288**	−0.261**	−0.382**	0.356**	1

***Indicates *p* < 0.001, **indicates *p* < 0.01, *indicates *p* < 0.05 (two-tailed tests).

### 4.3 Hypothesis testing

#### 4.3.1 Main effects and mediation effects testing

**Figure 2:**
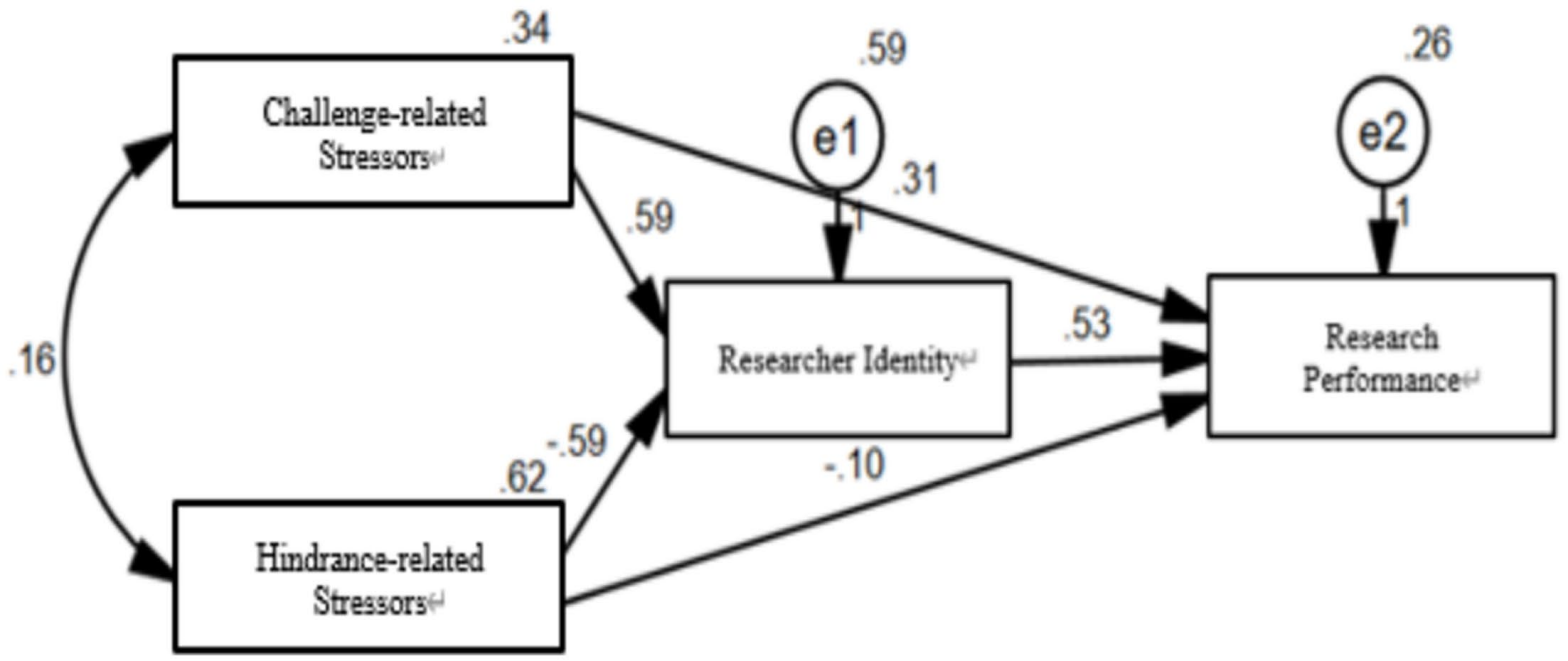
Standardized path coefficients of the structural equation model.

To explore the pathways between challenge- and hindrance-related stressors, researcher identity, and research performance, this study constructed a structural equation model. The model included challenge- and hindrance-related stressors as independent variables, research performance as the dependent variable, and researcher identity as the mediating variable, while controlling for demographic variables such as gender, age, institutional level, academic discipline, academic title, and educational background. Given that this model is saturated, where the number of estimated parameters equals the number of elements in the covariance matrix, the degrees of freedom are zero, and thus, no goodness-of-fit indices are estimated. Instead, the analysis focuses on the path coefficients, as depicted in Figure 2. The results indicate that challenge-related stressors have a significant positive effect on research performance (*β* = 0.31, *t* = 5.612, *p* < 0.001), and hindrance-related stressors have a significant negative effect on research performance (*β* = −0.10, *t* = −3.407, *p* < 0.05), confirming hypotheses *H1a* and *H1b*. Challenge-related stressors also positively influence researcher identity (*β* = 0.59, *t* = 4.302, *p* < 0.001), while hindrance-related stressors have a significant negative effect on researcher identity (*β* = −0.59, *t* = −3.706, *p* < 0.001), supporting hypotheses *H2a* and *H2b*. Furthermore, researcher identity has a significant positive effect on research performance (*β* = 0.53, *t* = 7.214, *p* < 0.001), supporting hypothesis *H3* and preliminarily verifying the mediating role of researcher identity.

This study further employed the bias-corrected non-parametric percentile bootstrap estimation method to test the mediation effect of researcher identity between challenge- and hindrance-related stressors and research performance, with 5,000 random resampling repetitions. If the 95% confidence interval does not include zero, the mediation effect is deemed significant. The results are presented in Table 2. First, the mediation effect of researcher identity between challenge-related stressors and research performance is 0.238, with a 95% confidence interval of [0.186, 0.403], indicating a significant mediation effect accounting for 49.90% of the total effect (0.477). Moreover, the mediation effect of researcher identity between hindrance-related stressors and research performance is −0.325, with a 95% confidence interval of [−0.411, −0.235], excluding zero, indicating a significant mediation effect, which accounts for 75.58% of the total effect (−0.43), thus confirming hypotheses *H4a* and *H4b*, see Table 4.

**Table 4:** The mediation analysis of research role identity.

Effect	Path	Effect value	SE	95%CI	*p*
Direct	Challenge stressors → research performance	0.239	0.078	[0.204, 0.425]	<0.001
	Hindrance stressors → research performance	−0.105	0.065	[−0.61, −0.361]	<0.05
Indirect	Challenge stressors → research role identity → research performance	0.238	0.135	[0.186, 0.403]	<0.001
	Hindrance stressors → research role identity → research performance	−0.325	0.081	[−0.411, −0.235]	<0.001
Total	Challenge stressors → research performance	0.477	0.009	[0.247, 0.553]	<0.001
	Hindrance stressors → research performance	−0.43	0.065	[−0.254, −0.078]	<0.001

#### 4.3.2 Moderating effects testing of research environment

To test the moderating role of the research environment between challenge- and hindrance-related stressors and research performance, the study first de-centered the three variables: challenge-related stressors, hindrance-related stressors, and the research environment. Interaction terms “challenge-related stressors × research environment” and “hindrance-related stressors × research environment” were then constructed. Multilevel linear regression was performed with research performance as the dependent variable, sequentially adding control variables, independent variables, moderating variables, and interaction terms between the independent and moderating variables. The regression results are shown in Table 5. The interaction term “challenge-related stressors × research environment” has a significant negative effect on research performance (*β* = −1.19, *t* = −2. 25, *p* < 0.001); similarly, the interaction term “hindrance-related stressors × research environment” has a significant negative effect on research performance (*β* = −2.90, *t* = −3.09, *p* < 0.001). It suggests that the research environment and challenge-related stressors have a substitution effect on research performance, meaning that when the research environment is relatively poor, the effect of challenge-related stressors on research performance is more pronounced; and when the research environment is relatively high, the effect of challenge-related stressors on research performance diminishes. At the same time, the research environment has a negative moderating effect on hindrance-related stressors. In a high-level research environment, high expectations and demands may exacerbate the negative effects of hindrance-related stressors. Overall, the research environment moderates the impact of challenge-related stressors on researcher identity and the impact of hindrance-related stressors on researcher identity, thus confirming hypotheses *H5a* and *H5b*.

**Table 5:** Regression analysis of moderating effects (*N* = 560).

Variable		Dependent variable: research performance						
		M1	M2	M3	M4	M5	M6	M7
Control variables	Gender	−0.02	−0.03	−0.07	−0.10	−0.01	−0.06	−0.08
	Age	−0.16*	−0.18*	−0.17**	−0.15*	−0.13	−0.15*	−0.15*
	Institutional level	−0.17**	−0.18**	−0.12*	−0.13*	−0.13*	−0.09	−0.09
	Academic discipline	−0.11	−0.11	−0.07	−0.06	−0.09	−0.07	−0.06
	Academic title	−0.34***	−0.34***	−0.33***	−0.32***	−0.30***	−0.32***	−0.31***
	Education level	−0.01	−0.04	−0.10	0.09	0.00	0.14	0.14
Independent variables	Challenge-related stressors		0.32***	0.25***	0.87***			
	Hindrance-related stressors					−0.19**	−0.07	0.77*
Moderating variable	Research environment			0.41***	1.33***		0.44***	1.15***
Interaction terms	Challenge-related stressors × research environment				−1.19***			
	Hindrance-related stressors × research environment							−0.94**
Parameters	*R* ^2^	0.11	0.22	0.35	0.38	0.15	0.29	0.31
	Adjusted *R*^2^	0.09	0.20	0.33	0.36	0.12	0.27	0.29
	Δ*R*^2^	0.11	0.10	0.13	0.03	0.03	0.14	0.02
	*F* change	5.75***	36.06***	53.15***	13.50***	10.64**	54.35***	8.43**

***Indicates *p* < 0.001, **indicates *p* < 0.01, *indicates *p* < 0.05 (two-tailed tests).

## 5 Discussion

### 5.1 Challenge-related stressors positively predict teachers’ research performance, while hindrance-related stressors negatively predict it

The study found that challenge-related stressors positively predict research performance, whereas hindrance-related stressors negatively predict it. This finding aligns with the challenge–hindrance framework and previous evidence (Lepine et al., 2005). One study also found that Challenge-related research stressors have a significant positive effect on research performance, whereas hindrance-related research stressors have a significant negative effect (Wang et al., 2014). Another study examined how research stress influence young teachers’ research performance, and divided the research stress into information cognition and control cognition of scientific research stress based on cognitive evaluation theory. They found that informational stress cognition promotes performance, whereas controlling cognition impairs it (Liu and Yu, 2018). This study offers valuable insights into the subjective interpretation of stress which is consistent with the results of this study to some extent. However, it does not distinguish between the objective nature of stressors (challenge vs. hindrance). In contrast, the present study adopts the challenge–hindrance stressor framework, which focuses on the objective characteristics of stressors rather than subjective appraisal alone. A plausible explanation lies in the unique context of Chinese higher education: the “Double First-Class” policy and “publish or perish” culture have raised baseline pressure to an extremely high level, potentially pushing many teachers beyond the optimal point of the Yerkes-Dodson inverted-U curve. Thus, what might be purely challenging in less demanding environments could partially transform into hindrance under excessive strain (Crawford et al., 2010). Moreover, according to Self-Determination Theory (Deci and Ryan, 1985), individuals experience intrinsic motivation and self-efficacy when facing challenges accompanied by autonomy. Yet, in many Chinese universities, high-stakes evaluation systems often limit perceived autonomy, which may attenuate the motivational benefits of challenge stressors. The Yerkes-Dodson Law further suggests that moderate challenge stress optimizes arousal and performance (Corbett, 2015). Our results imply that many teachers may already operate near or beyond the optimal level, explaining why the positive effect, while significant, is not larger.

### 5.2 Researcher identity mediates the relationship between challenge-and hindrance-related stressors and teachers’ research performance

Our results indicate that researcher identity differentially mediates the effects of challenge and hindrance stressors on research performance. Specifically, challenge-related stressors enhance researcher identity, which in turn promotes teachers’ research performance. In contrast, hindrance-related stressors diminish researcher identity, thereby inhibiting research performance. These findings support Role Identity Theory (Burke and Reitzes, 1991), which posits that role identity originates from the self-concept formed through interactions between individuals and their role environments, and subsequently influences role behaviors. Positive interactions—such as overcoming challenges, receiving recognition, or achieving mastery—strengthen identity, whereas negative interactions—such as repeated failures, unfairness, or resource deprivation—weaken it (Anglin et al., 2022). The results also align with Conservation of Resources Theory (Hobfoll, 1989): challenge stressors provide resource gains (self-efficacy, achievement) that enhance identity, while hindrance stressors cause resource depletion (energy, time, emotional stability) that undermines identity. A related study examined the impact of challenge–hindrance research stressors on knowledge sharing behavior, also identifying research role identity as a mediator. That study found that challenge stressors positively influenced knowledge sharing via enhanced researcher identity, whereas hindrance stressors negatively affected knowledge sharing through diminished identity. These findings are largely consistent with our results, suggesting that researcher identity serves as a robust psychological mechanism transmitting the effects of both types of stressors to different outcome variables—namely, research performance (in our study) and knowledge sharing behavior (in the related study). Importantly, the mediation is only partial, indicating that other psychological mechanisms like work engagement, psychological capital, emotional exhaustion also operate in parallel. This suggests that future research should explore additional mediators and examine potential moderators that may strengthen or weaken the identity pathway. From a practical standpoint, universities can strengthen teachers’ researcher identity through mentoring programs, academic recognition, and creating a sense of academic belonging can further amplify the positive effects of challenge stressors while buffering the negative impact of hindrance stressors. Overall, these results underscore the importance of understanding how different types of research pressure operate through identity to shape performance, offering both theoretical insights and actionable guidance for university research management.

### 5.3 Research environment moderates the relationship between challenge– and hindrance-related stressors and teachers’ research performance

This study found that the research environment, as a supportive factor, moderates the relationship between challenge and hindrance-related stressors and teachers’ research performance in different ways. On one hand, the research environment and challenge stressors act as substitutes. Specifically, for teachers in highly supportive research environments, the positive effect of challenge stressors on research performance is weaker, because environmental resources already stimulate intrinsic motivation, leaving less marginal gain from additional challenges. Conversely, for teachers in less supportive environments, challenge stressors play a more important compensatory role, driving research performance through individual effort rather than institutional support. On the other hand, even abundant environmental resources may not fully eliminate the negative effects of hindrance stressors. In high-level research environments, elevated expectations and demands may even exacerbate these negative effects. According to Conservation of Resources Theory, persistent hindrance stressors continuously deplete teachers’ psychological and physical resources (Hobfoll, 1989). So, it is advisable to avoid overburdening high-support research environments with excessive performance expectations. Although high-level academic settings frequently impose stringent publication quotas and recurrent evaluations, such practices may paradoxically exacerbate resource depletion. To mitigate this, universities should decouple resource support from punitive performance pressure by adopting formative evaluation systems that emphasize research processes and long-term scholarly contributions. Moreover, institutional support should be extended to incorporate psychological and humanistic care, as current systems predominantly focus on funding and career advancement while neglecting mental health, work-life balance, and individual development. Establishing counseling services, stress management training programs, and peer mentoring networks constitutes an effective approach to addressing these gaps.

## 6 Conclusion

This study constructed a mediation model and a moderation model to examine the mediating role of researcher identity between challenge– and hindrance-related stressors and teachers’ research performance, as well as the moderating role of the research environment. The results indicate that different types of research stressors exert distinct influences on research performance. Specifically, researcher identity mediated the relationship between challenge and hindrance-related stressors and research performance, while the research environment moderated the relationship. These findings suggest that teachers’ research performance is enhanced through the interaction between external research environments and internal research cognitions. This study thus provides theoretical insights into teachers’ research psychology and offers practical guidance for promoting their research performance.

## 7 Practical implications

Based on the above findings, this study proposes the following countermeasures to promote teachers’ research performance:

### 7.1 Develop research incentive and support mechanisms aimed at enhancing research self-efficacy by differentiating between different types of research stressors

Universities should set moderate, SMART challenge-oriented goals to stimulate motivation while avoiding excessive pressure that turns challenges into hindrances. Individualized incentives based on teachers’ abilities and interests are keys. For hindrance stressors (e.g., vague evaluation standards, resource shortages, research isolation), universities should develop clear evaluation systems, increase research resources, and foster collaborative research teams to reduce negative impacts and sustain self-efficacy and role identity.

### 7.2 Enhance teachers’ researcher identity to improve their research performance

Challenge stressors enhance researcher identity; hindrance stressors undermine it^[57].^ Universities should encourage teachers to set challenging but achievable goals to boost achievement and identity. They should also help teachers reduce hindrance stressors. and provide conflict resolution and stress management programs. According to the JD-R model, adequate resources buffer stress and strengthen role identification. Thus, universities should build a supportive research environment—particularly soft aspects like research culture that tolerates failure and encourages innovation. This helps teachers internalize role expectations into self-concept, enhancing motivation. As confirmed by this study, stronger researcher identity leads to greater investment in academic activities and improved research performance.

### 7.3 Provide adequate research resources to support targeted and effective resource allocation

High-level research environments do not fully eliminate hindrance stressors, as these stressors stem from cumbersome tasks, resource shortages, unfriendly climates, and unreasonable evaluations. Prolonged exposure depletes teachers’ resources, reducing efficiency and outcomes. Moreover, high-level environments often bring greater competition and higher demands, which may amplify hindrance effects. However, this does not mean improving research support is useless. Universities should enhance the targeting and effectiveness of resource allocation—not just provide resources but also optimize management systems. Specific measures include: adopting diversified evaluation criteria (beyond publication counts), promoting collaborative research teams to share pressure and build cohesion, and simplifying administrative paperwork. These actions can optimize the research environment and improve teachers’ research efficiency and outcomes.

## 8 Limitations and future research directions

First, this study employed cross-sectional data, which limits the ability to infer causal relationships between research stressors and research performance. Although statistical analyses revealed correlations between variables, future research could adopt longitudinal designs and time-point measurements to clarify the causal paths of challenge- and hindrance-related stressors on research performance, thereby enhancing the reliability of the findings. Second, while this study examined the interaction between the research environment and research stressors, it did not delve into the specific factors within the research environment (such as resource support and academic culture) and their mechanisms under different stressors. Future research could further decompose the various dimensions of the research environment to elucidate the complexities of how they influence challenge- and hindrance-related stressors. Additionally, integrating different levels of environmental factors could provide a more comprehensive understanding of the role of the research environment in research performance.

## Data Availability

The raw data supporting the conclusions of this article will be made available by the authors, without undue reservation.
